# Facile and High-yield Synthesis of N-doped Carbon Quantum Dots from Biomass Quinoa Saponin for the Detection of Co^2+^

**DOI:** 10.1155/2021/9732364

**Published:** 2021-12-23

**Authors:** Cuo Zhou, Shunwei Wu, Shenghui Qi, Weijun Song, Chunyan Sun

**Affiliations:** School of Chemical Engineering, Qinghai University, Xining 810016, China

## Abstract

Hydrothermal synthesis of carbon quantum dots (CQDs) from natural biomass is a green and sustainable route for CQDs applications in various fields. In this work, the preparation and characterization of CQDs based on quinoa saponin were investigated. The optimum synthetic conditions determined by orthogonal experiments were as follows: 2 g quinoa saponin powder and 0.04 mol ethylenediamine reacted at 200°C for 10 h. The relative fluorescence quantum yield (QY = 22.2%) can be obtained, which is higher than some results reported in the literatures. The prepared CQDs had a small and uniform size (∼2.25 nm) and exhibited excitation wavelength-dependent blue light emission behavior. The CQDs displayed excellent sensitivity for Co^2+^ detection along with good linear correlation ranging from 20 to 150 *µ*M and the detection limit of 0.49 *µ*M. The CQDs prepared in this experiment were successfully implanted into soybean sprouts for fluorescence imaging. The sprouts could grow healthily even soaked in the CQDs solution for two weeks, demonstrating the low toxicity of the CQDs. The advantages of the CQDs, such as low cost, ease of manufacture, nontoxicity, and stability, have potential applications in many areas such as metal ion detection and biosensing.

## 1. Introduction

As an important component of vitamin B_12_, cobalt promotes human bone marrow hematopoiesis and hemoglobin synthesis and increases the number of red blood cells [1]. Studies have shown that an excess or deficiency of cobalt in the human body can cause various diseases, so the detection of cobalt ions in the human body is of great importance [2]. Currently, there are more methods to determine cobalt ions, such as mass spectrometry [3], atomic absorption [4], and electrochemical methods [5]. Although these methods can detect trace amounts of cobalt ions, they still have some disadvantages, such as complicated test operations, time-consuming, and expensive test instruments. The fluorescence detection method has attracted increasing attention due to its simplicity, good sensitivity with selectivity, faster response time, minimal interference effect, and relatively low-cost.

Carbon quantum dots (CQDs), as one new type of nano-materials, are usually spherical in shape and with the size below 10 nm. CQDs have attracted more and more attention due to their excellent properties, including good fluorescence characteristics, water solubility, bio-compatibility, and low toxicity. Based on the excellent properties, CQDs were widely applied in the fields of detection, drug delivery, and bioimaging [6–9]. The emergence of CQDs has enriched the application scope of this class of carbon nanomaterials, but the problems of complex postprocessing and low yield still affect their practical applications. Therefore, the development of carbon sources with abundant sources, low prices, and high yields has been a matter of great interest.

Among numerous precursors for the synthesis of CQDs, natural biomass is the most promising one due to its low cost, renew ability, abundance, and environmental friendliness. Some daily life foods or crops (coffee [10], garlic [11], grass [12], highland barley [13], shrimp shells [14], etc.) have been used as carbon source materials for the preparation of CQDs, but these CQDs often possess relatively low fluorescence quantum yield (less than 10%). Quinoa, an upland specialty crop, is rich in high-quality protein, carbohydrates, and dietary fiber. The seed coat of quinoa is covered with a water-soluble, bitter, and antinutritional saponin [15, 16], which are required to be removed before eating. Therefore, the approaches through which the saponin from quinoa processing industry may be utilized have become a consideration.

In this paper, CQDs with high relative fluorescence quantum yields (22.2%) and good water solubility were prepared by a one-step hydrothermal method using biomass waste quinoa saponins as a carbon source. The reaction is safe and inexpensive in terms of raw materials, simple in process, and does not require catalysts, and so on. Meanwhile, a rapid detection method of Co^2+^ was established using the fluorescence quenching of Co^2+^ on the CQDs. The use of biological waste as raw materials is energy-efficient and environmentally friendly, and does not contain toxic components such as metal ions, which is more beneficial for its application in the biological field.

## 2. Materials and Methods

### 2.1. Chemicals and Reagents

Quinoa saponin was provided by Gaoyuan Jinhe Ecological Agriculture and Animal Husbandry Technology Co., Ltd, Xining, China, smashed before use. CuCl_2_, MgCl_2_, FeCl_3_, NiCl_2_, Co(NO_3_)_2_, Pb(NO_3_)_2_, CrCl_3_, CdCl_2_, ZnCl_2_, HgCl_2_, ethylenediamine (EDA), carbamide (Urea), aqueous ammonia (NH_4_OH, 25%), and diethanolamine (DEA) were purchased from Aladdin Biochemical Technology Co., Ltd, Shanghai, China. Soybean seeds were purchased from retail outlets. All the reagents were of analytical reagent grade and used without further purification. Ultrapure water was used during the entire experimental process.

### 2.2. Apparatus

The morphology and microstructure of CQDs were verified by high-resolution transmission electron microscope (Tecnai G2 F20, FEI Inc., America). Samples were prepared by dropping an aqueous solution onto a 300-mesh copper grid deposited with a carbon film, accelerated voltage: 200 kV. X-ray powder diffraction (XRD) patterns were obtained on a D/Max 2500 PC X-ray diffractometer (Rigaku, Kyoto, Japan) with Cu K*α* radiation. The chemical composition of CQDs was obtained with an X-ray photoelectron spectrometer (TM ESCALABTM 250Xi, Thermo Scientific, Waltham, MA, USA) using an excitation source of Al K*α* rays (1486.6 eV). The infrared spectra in KBr were obtained using an FT-IR BXII spectrometer (Perkin-Elmer, Waltham, MA, USA). The UV/Vis spectra were recorded on a spectrophotometer (T6-general analysis, Beijing, China). Fluorescent measurements were performed on the FL-7000 fluorescence spectrophotometry (Hitachi, Tokyo, Japan). Photographs were taken using a Dark box type four-purpose ultraviolet analyzer (WFH-203C, Shanghai, China). The sterilized water for culturing bean sprouts was obtained by treating ultrapure water in an autoclave (KG-SX-500, Kaogoshima SEISAKUSYO Inc., Niigata, Japan). The carbon quantum dot powder was obtained by drying in a vacuum freeze dryer (Zhejiang Xinzhi Biotechnology Co., Ltd., Ningbo China).

### 2.3. Hydrothermal Synthesis of CQDs

The CQDs were synthesized using biomass quinoa saponin powder as carbon source and ethanediamine as nitrogen source through a green hydrothermal method. The optimum synthetic conditions determined by orthogonal experiments were shown below, 2.0 g quinoa saponin powder and 0.04 mol (2.668 mL) ethanediamine were poured into ultrapure water (30 mL). The mixture was ultrasonicated for 10 min, and sealed in a 50 mL reaction kettle lined with Teflon and heated at 200°C in an oven for 10 h. After cooling to room temperature, the product was centrifuged at 9500 rpm for 15 min, following supernatant dialysed against ultrapure water for 12 h by a dialysis membrane (MWCO = 500 D). Finally, the solution was dried under vacuum freeze-dryer until a dark brown product was obtained. CQDs without N doping were prepared under the same conditions (no N dopant was added during the preparation).

### 2.4. Fluorescence (FL) Measurements

The FL measurements were performed under the following conditions: the slit widths of the incident wavelength and emission wavelength were 2.5 nm and 5.0 nm, respectively. A scan rate of 12000 nm/min, and a photomultiplier tube voltage of 400 V were used. The change in FL intensity of the corresponding emission wavelength was used to determine the optimal excitation wavelength. The effects of the concentration and pH of the CQDs solution on the FL properties were studied separately.

The FL titration experiments for the detection of Co^2+^ by CQDs were carried out: different concentrations of Co^2+^ solutions were prepared in the range of 0∼200 *μ*M. Next, 3 mL of CQDs solution was added to the cuvette, followed by 1 mL of Co^2+^ solution; after standing for 10 min, the FL intensity of the mixture was recorded at the excitation wavelength of 390 nm. All FL experiments were carried out at room temperature.

### 2.5. Measurements of FL Quantum Yield

The quantum yield (QY) of CQDs was examined based on a comparative method using quinine sulfate as the reference. Briefly, CQDs were diluted with ultrapure water (*η* = 1.33), and quinine solution as a standard was diluted in 0.1 M H_2_SO_4_ (QY = 54%, *η* = 1.33). The absorbance and FL spectra were recorded at 366 nm. The absorbance values of CQDs and quinine solutions were maintained in the range of 0.08∼0.1 in a 1 cm quartz cuvette. The FL intensity obtained for the sample as a function of absorbance was compared to that of quinine using the equation:

(1) $$\begin{array}{c} QY_{x}=QY_{s}\times \frac{A_{s}}{A_{x}}\times \frac{F_{x}}{F_{s}}\times \frac{\eta_{x}^{2}}{\eta_{s}^{2}}, \end{array}$$

where “*A*” is the absorbance at 366 nm, “*F*” is the FL emission curve integrated area, “*η*” is the solvent refraction index, “*x*” refers to CQDs, and “*s*” refers to quinine.

### 2.6. FL Bioimaging

The soybean seeds were used to grow sprouts for the bioimaging to evaluate the biological toxicity. Two groups of seeds (test group and control group) were used. The soybean seeds were sterilized by soaking in 30% H_2_O_2_ for 10 min and rinsed 5–7 times with deionized water. The treated soybean seeds were placed in Petri dishes soaked with ultrapure water for 12 h and then transferred to paper beds. The seeds were incubated in the dark at 20°C for 48 h sprouting. Sterilized water was employed during the breeding process. After 48 hours of incubation, sterilized water was replaced with the CQDs solution (0.8 mg/mL) for the test group. After another 72 hours, the soybean sprouts were reaped, followed by washing with sterilized water to remove the residual agents. The two groups soybean sprouts were irradiated with UV lamp (365 nm) and photographed, respectively, to record the FL imaging.

## 3. Results

### 3.1. Optimization of Synthesis Conditions

**Figure 1:**
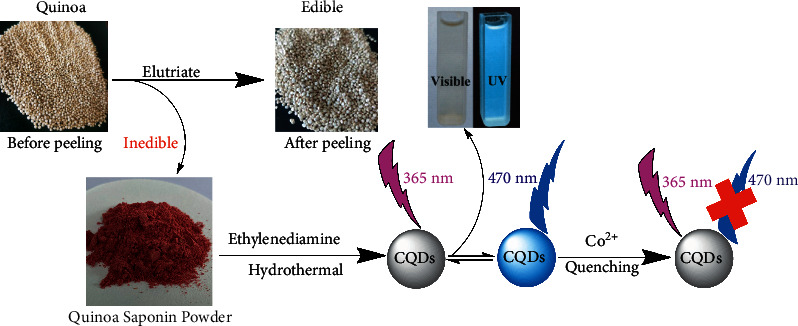
Schematic illustration of synthesis route and application for Co^2+^ detection.

In this study, the CQDs were easily synthesized by a hydrothermal method from quinoa saponin. It is widely accepted that hydrothermal carbonization of biomass is a green method for the preparation of CQDs [17]. However, the FL properties of CQDs were largely influenced by preparation conditions, which need to be further optimized. The FL quantum yield (QY) of CQDs was used as a criterion to find the optimal synthesis conditions. We determined the optimal preparation condition by designing orthogonal experiments (see supplementary materials for details), which gives the result as follows: 2 g quinoa saponin powder (carbon source) and 0.04 mol ethylenediamine (N-dopant) reacted at 200°C for 10 h. Under the optimized conditions, the quantum yield of CQDs can reach 22.2%, which is higher than that of reported biomass carbon point as shown in Table 1. The as-prepared CQDs in aqueous solution emit blue light under the irradiation of UV light (365 nm) as shown in Figure 1. The follow-up studies showed that Co^2+^ could sensitively quench the FL of the CQDs solution, and the CQDs could be used for quantitative detection of Co^2+^.

### 3.2. Characterization of CQDs

**Figure 2:**
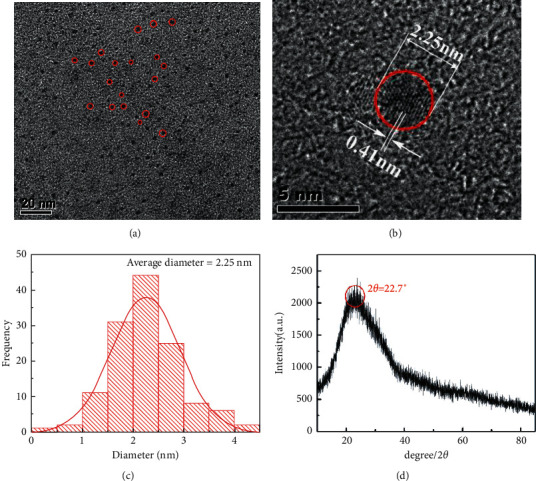
(a) TEM image of CQDs; (b) HRTEM image of CQDs; (c) Diameter distribution of CQDs; (d) XRD pattern of CQDs.

The microstructure of CQDs was investigated by TEM as shown in Figure 2(a), where the shape and monodispersion of CQDs are almost close to quasi-spherical. The HRTEM image in Figure 2(b) shows a crystalline surface spacing of 0.41 nm, corresponding to the graphitic amorphous structure. The CQDs have a uniform diameter ranging from 1.5 to 3.0 nm with an average diameter of 2.25 nm (Figure 2(c)). This result confirmed the successful synthesis of small spherical CQDs. The XRD pattern of CQDs in Figure 2(d) shows a broad diffraction peak at 2*θ* = 22.7°, and the layer spacing *d* can be calculated from the Bragg formula to be 0.39 nm, which is larger than that of graphite (0.34 nm) [23]. The increase of the *d* value indicates an increase in the amorphous nature, which can be attributed to the introduction of more defect sites into the product by increasing the N doping of the lattice [13].

**Figure 3:**
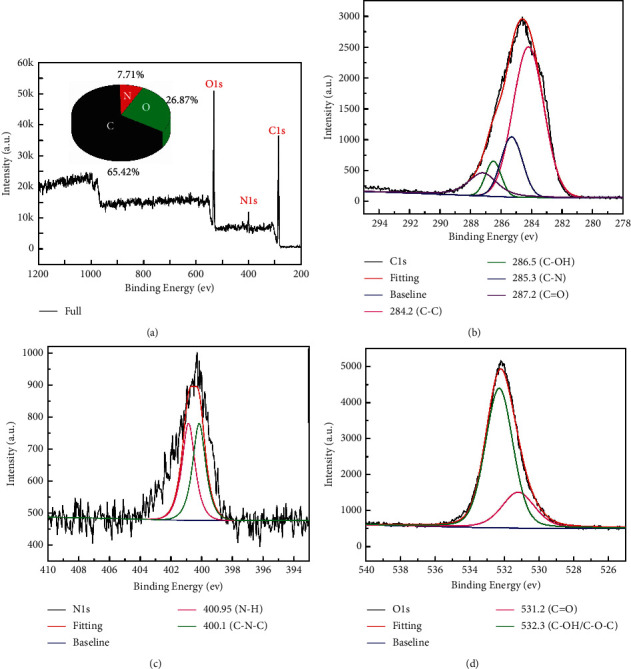
(a) XPS full spectrum of CQDs; (b–d): C1s, N1s, and O1s peaks of high-resolution XPS spectra, respectively.

XPS was performed to analyze the surface elements and their binding states of CQDs. As shown in Figure 3(a), the CQDs show three main strong peaks at 285.68, 400.32, and 531.82 eV, attributed to C1, N1 and O1 electron binding energies, respectively [24]. The contents of C, N, and O were calculated to be 65.42%, 7.71%, and 26.87%. The high-resolution spectra of C1s exhibit four main peaks at 284.2, 285.3, 286.5, and 287.2 eV (Figure 3(b)), which were assigned to C-C/C=C, C-N, C-O and C=O bonds, respectively [18, 25]. In the N1s spectrum shown in Figure 3(c), the two peaks at 400.1 eV and 400.95 eV were attributed to the C-N-C and N-H bonds, respectively [26]. From the O1s spectrum shown in Figure 3(d), the two fitted peaks at 531.2 and 532.3 eV were attributed to the C=O and C-OH/C-O-C bonds, respectively [27].

**Figure 4:**
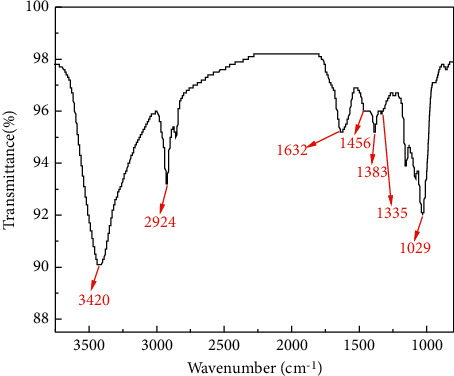
The FT-IR spectrum of the CQDs.

FT-IR spectra in Figure 4 were also used to verify the functional groups of the synthesized CQDs. The characteristic absorption band at 3420 cm^−1^ corresponds to the stretching vibrations of the O-H and N-H bonds [28, 29]. The peak at 2924 cm^−1^ and 1335 cm^−1^ were attributed to the stretching vibration of the C-H bond [30]. The peaks at 1632 cm^−1^ and 1456 cm^−1^ indicate the presence of C=O and C=C, respectively [31]. The characteristic absorption band of C-N was also observed at 1383 cm^−1^, indicating the successful doping of N atoms in CQDs [32]. In addition, a broad band near 1029 cm^−1^ was attributed to the bending vibrations of C-O [33]. The conclusive evidence from FT-IR and XPS data can confirm that the synthesized CQDs have a large number of hydrophilic groups such as hydroxyl (-OH), carboxyl (-COOH), and amide (-NH_2_) portions, which could improve the stability of CQDs in aqueous solution.

### 3.3. Optical Properties of the CQDs

**Figure 5:**
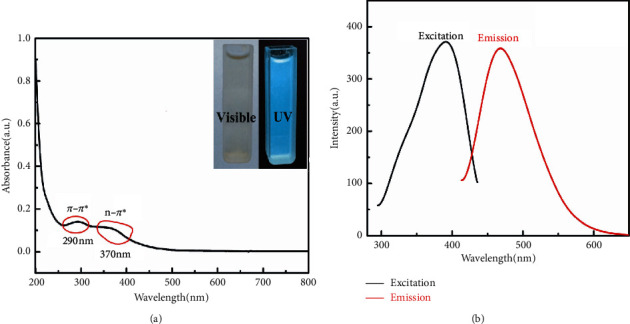
(a) UV-vis absorption spectra of CQDs; (b) FL spectra of CQDs.

UV-vis absorption and FL spectrum were carried out in details. As illustrated in Figure 5(a), CQDs solution exhibit a broad UV absorption band with characteristic absorption near 290 nm and 370 nm, resulting from the *π*-*π*^*∗*^ leap of the C=C group and the *n*-*π*^*∗*^ leap of the C=O group, respectively [34, 35]. Further, the inset of Figure 5(a) shows the photographic image of CQDs aqueous solution under visible light and UV-light (365 nm), indicating that the prepared CQDs have strong FL. It can be seen from the emission spectra of CQDs (Figure 5(b)), the maximum FL emission wavelength of CQD is 470 nm when excited at 390 nm. Thus, an excitation wavelength at 390 nm was used in further experiments.

**Figure 6:**
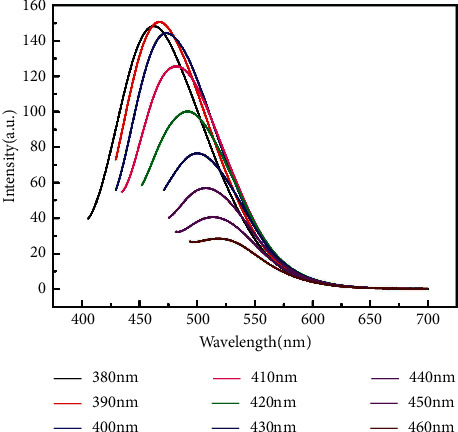
FL spectra of CQDs at different excitation wavelengths.

Figure 6 shows the excitation-dependent emission spectra of the synthesized CQDs. When the excitation wavelengths changed from 380 to 460 nm, the emission wavelength was red-shifted from 460 to 520 nm. The strongest emission was obtained at 390 nm excitation, and the FL intensity gradually decreased with the excitation wavelengths from 390 to 460 nm. This phenomenon may be caused by the incomplete dimensional uniformity of the CQDs and some functional groups on the surface, or the various surface energy traps [36, 37].

### 3.4. Effects of the Concentration and pH on CQDs Fluorescence

**Figure 7:**
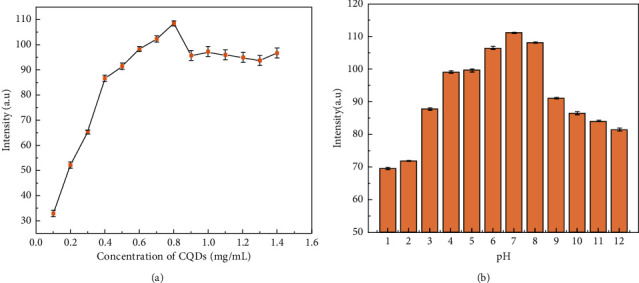
(a) FL intensity of CQDs at different concentrations; (b) FL intensity of CQDs at different pH.

The effect of concentration on the CQDs FL intensities was studied in the range of 0.1–1.4 mg/mL. As shown in Figure 7(a), the intensity increased with increasing the CQDs concentration from 0.1 to 0.8 mg/mL, and then decreased slightly when the concentration exceeded 0.8 mg/mL. The decrease of FL intensity at high concentration may be caused by the FL self-absorption phenomenon. Therefore, in the next experiments, the concentration of CQDs was chosen to be 0.8 mg/mL.

The pH is one of the important physicochemical parameters affecting the application of CQDs. The effect of the pH of CQDs solution on FL intensity is depicted in Figure 7(b). The FL intensity is low when the solution pH is less than 3 or higher than 9, and the FL intensity reaches the highest value when the pH is 7. The decrease of FL intensity under alkaline conditions may be caused by the agglomeration of CQDs. Under strong acidic and alkaline conditions, CQDs are relatively unstable due to the large number of carboxyl groups on the surface of CQDs, which are prone to hydrogen bonding and agglomeration between CQDs under acidic conditions, making the fluorescence of carbon quantum dots quenching. Under neutral conditions, CQDs dissociate and are mono-disperse, so the fluorescence intensity is high and remains stable, which may also be due to the change of surface charge caused by the protonation and deprotonation of CQDs [38]. Therefore, the pH of the solution was adjusted to 7 in the next experiments to obtain the best FL intensity.

### 3.5. Selectivity of CQDs toward Metal Ions

**Figure 8:**
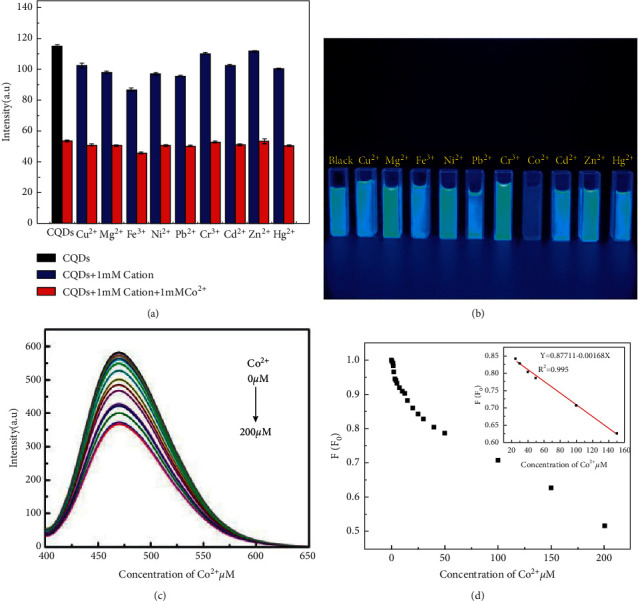
(a) The selectivity test of CQDs (0.8 mg/mL) for Co^2+^ against different metal ions (excitation wavelength = 390 nm); (b) FL photographs of CQDs with various metal ions under the UV-lamp (*λ* = 365 nm); (c) FL emission spectra of the CQDs (0.8 mg/mL) upon exposure to various concentrations of Co^2+^ (from top to bottom: 0, 1, 2, 3, 4, 6, 8, 10, 15, 20, 25, 30, 40, 50, 60, 80, 100, 200 µM) in aqueous solution excitation at 390 nm; (d) The calibration curves and linear equation for the FL quenching ratio and concentration of Co^2+^.

To investigate the selectivity of the CQDs, nine kinds of other biological and environmental relevant metal ions (including Cu^2+^, Mg^2+^, Fe^3+^, Ni^2+^, Pb^2+^, Cr^3+^, Cd^2+^, Zn^2+^, Hg^2+^, 1 mM) were added to the solution of CQDs (0.8 mg/mL), When these ions were respectively added in the solution of CQDs, no obvious FL intensity change occurred, as shown in Figure 8(a). However, after injecting Co^2+^ (1 mM) into CQDs solution containing other metal ions, Co^2+^ could validly quench the FL of CQDs solution.  Figure 8(b) also shows a remarkable FL quenching for only Co^2+^ compared with the other metal cations. This result proved the highly selectivity of CQDs for Co^2+^ over other competitive metal ions. The FL selectivity for metal ions was determined based on their FL intensity at pH = 7 (0.1 mol/L HCl and 0.1 mol/L NaOH were used to adjust the pH of the solution).

The relationship between the FL intensity of the CQDs and Co^2+^ concentration (0 to 200 *μ*M) was measured by Co^2+^ titration experiments, and the results are shown in Figure 8. From the Figure 8(c), it can be seen that the FL intensity decreased regularly with the increase of Co^2+^ concentration, and FL quenching did not cause the shift of emission peak.  Figure 8(d) shows the calibration curve obtained for Co^2+^ concentration. FL quenching ratio F/F_0_ (F_0_ denotes the initial FL intensity of the solution without the addition of Co^2+^, and F denotes the FL intensity after the addition of Co^2+^). As shown, there was a good linear correlation between the quenching efficiency against concentration of Co^2+^ in the range from 20 to 150 *μ*M (*R*^2^ = 0.995). According to three times the standard deviation regulation [39], the limit of detection (LOD) was calculated as 0.49 *μ*mol/L.

### 3.6. Imaging of CQDs in Bean Sprouts

**Figure 9:**
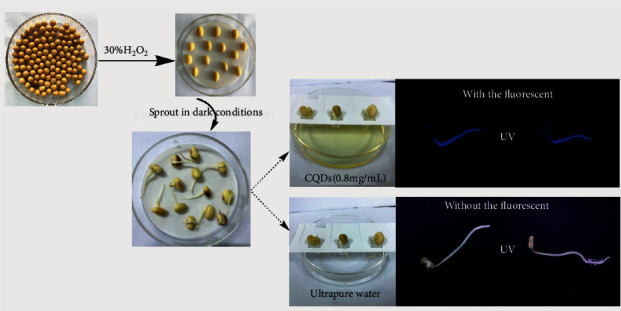
Soya bean sprout cultivation process and the images of the sprouts under UV (365 nm).

Herein, soybean sprouts were employed as the fluorescence matrix to evaluate the biological toxicity and fluorescence bioimaging of CQDs.  Figure 9 shows the cultivation process of soybean sprouts. The sprouts could grow up healthily after the addition of the CQDs solution, even two weeks later, demonstrated the low cytotoxicity of the as-prepared CQDs. In addition, randomly selected soybean sprouts cultivated with CQDs solution all emitted obvious blue fluorescence under the excitation of UV, while those cultivated in blank control group were not. Thus, the CQDs prepared in this experiment were successfully implanted into soybean sprouts with fluorescence imaging and little effect on their growth. The low toxicity, excellent biocompatibility, and good water-solubility make it expected to be used as biological probe.

## 4. Conclusions

Herein, we used an economic, simple, and green method to produce a water-soluble CQDs by hydrothermal treatment using biomass waste quinoa saponin as a carbon source. The synthesis conditions were optimized by orthogonal experiments, and obtained the CQDs with higher QY (22.2%). The CQDs exhibited a bright blue with FL emission under UV light irradiation. The degree of fluorescence quenching showed a good linear relationship with Co^2+^ concentration in the range of 20–150 *µ*M, with *R*^2^ = 0.995 and the detection limit of 0.49 *µ*M. Furthermore, the photostability and low toxicity of the CQDs successfully enable their application in bioimaging. We believe that this study has made an improvement in detection cost and accuracy. The proposed method could contribute to the research of CQDs as fluorescent sensors for metal ion detection and bioimaging.

## Figures and Tables

**Table 1:** Comparison of the proposed CQDs with various reported biomass carbon points.

Carbon source	Preparation method	QY (%)	References
Apple juice	Hydrothermal	4.3	[18]
Cornflour	Hydrothermal	7.7	[19]
Phyllanthus acidus fruits	Hydrothermal	14.0	[20]
Garlic	Hydrothermal	10.5	[11]
Watermelon peel	Pyrolytic carbonization	7.1	[21]
Lychee seeds	Pyrolytic carbonization	10.6	[22]
Quinoa saponin	Hydrothermal	22.2	This work

## Data Availability

The data used to support the findings of this study are included within the article, and any further information is available from the corresponding author upon request.

## Supplementary Materials

Supplementary Materials The optimal preparation conditions for CQDs determined by the orthogonal experiments are detailed in the supplementary materials. Table S1: the factors and levels of orthogonal experiment (basic conditions). Table S2: the orthogonal experiment scheme (basic conditions). Table S3: the numerical of orthogonal tests (basic conditions). Table S4: the factors and levels of orthogonal experiment (doping conditions). Table S5: the orthogonal experiments scheme (doping conditions). Table S6: the numerical of orthogonal tests (synthetic conditions). Figure S1: repeatability test of synthesis conditions.
