# Supplementary material for: Facile and High-yield Synthesis of N-doped Carbon Quantum Dots from Biomass Quinoa Saponin for the Detection of Co2+
Source: J Anal Methods Chem. 2021 Dec 23;2021:9732364. doi: 10.1155/2021/9732364 (PMC8718314; doi:10.1155/2021/9732364)
Supplement: Supplementary Materials — The optimal preparation conditions for CQDs determined by the orthogonal experiments are detailed in the supplementary materials. Table S1: the factors and levels of orthogonal experiment (basic conditions). Table S2: the orthogonal experiment scheme (basic conditions). Table S3: the numerical of orthogonal tests (basic conditions). Table S4: the factors and levels of orthogonal experiment (doping conditions). Table S5: the orthogonal experiments scheme (doping conditions). Table S6: the numerical of orthogonal tests (synthetic conditions). Figure S1: repeatability test of synthesis conditions. [file 9732364.f1.docx]

**Supplementary Materials**

# Facile and High-yield Synthesis of N-doped Carbon Quantum Dots from Biomass Quinoa Saponin for the Detection of Co^2+^

Cuo Zhou,^1^ Shunwei Wu,^1^  Shenghui Qi,^1^ Weijun Song,^1^ and Chunyan Sun^1^

^1^ School of Chemical Engineering, Qinghai University, Xining 810016, China.

Correspondence should be addressed to Chunyan Sun; sunchunyan@qhu.edu.cn

To achieve a high Fluorescence (FL) quantum yield of CQDs, the synthetic conditions were optimized by orthogonal experiments. The FL quantum yield (QY) of CQDs was used as a criterion to find the optimal synthesis conditions.

1. ***Optimization of basic conditions***

Taking the FL quantum yield of CQDs as the index, the 3-factor (quinoa saponin powder dosage, hydrothermal temperature and hydrothermal time), 4-level orthogonal experiments were designed firstly, as shown in Table S1. The orthogonal table L_16_(4^3^) as shown in Table S2 was used to conduct the orthogonal experiments without considering the interaction of the factors. According to the orthogonal experimental data analysis method, a total of 16 sets of orthogonal experiments were designed, for which the FL quantum yields were measured, and the results are shown in Table S3. The results of data analysis showed that the optimum basic conditions should be: quinoa saponin powder dosage of 2 g, hydrothermal temperature of 200 °C, and hydrothermal time of 10 h.

**Table S1.** The factors and levels of orthogonal experiment (basic conditions).

| Level  Factors | A  Amount of quinoa saponin powder / g | B  Temperature / °C | C  Time / h |
| --- | --- | --- | --- |
| 1 | 1 g | 140 °C | 6 h |
| 2 | 2 g | 160 °C | 8 h |
| 3 | 3 g | 180 °C | 10 h |
| 4 | 4 g | 200 °C | 12 h |

**Table S2.** The orthogonal experiment scheme (basic conditions).

| Test number  Factors | A  Amount of quinoa saponin powder / g | B  Temperature / °C | C  Time / h |
| --- | --- | --- | --- |
| 1 | 1 g | 140 °C | 6 h |
| 2 | 1 g | 160 °C | 8 h |
| 3 | 1 g | 180 °C | 10 h |
| 4 | 1 g | 200 °C | 12 h |
| 5 | 2 g | 140 °C | 8 h |
| 6 | 2 g | 160 °C | 6 h |
| 7 | 2 g | 180 °C | 12 h |
| 8 | 2 g | 200 °C | 10 h |
| 9 | 3 g | 140 °C | 10 h |
| 10 | 3 g | 160 °C | 12 h |
| 11 | 3 g | 180 °C | 6 h |
| 12 | 3 g | 200 °C | 8 h |
| 13 | 4 g | 140 °C | 12 h |
| 14 | 4 g | 160 °C | 10 h |
| 15 | 4 g | 180 °C | 8 h |
| 16 | 4 g | 200 °C | 6 h |

**Table S3.** The numerical of orthogonal tests (basic conditions).

| Test number | Experimental combinations | QY / % | k_Ai_ _(i=1-4)_ | k_Bi (i=1-4)_ | k_Ci (i=1-4)_ |
| --- | --- | --- | --- | --- | --- |
| 1 | 1 g-140 °C-6 h | 1.32 % | 2.23 % | 2.01 % | 1.80 % |
| 2 | 1 g-160 °C-8 h | 1.58 % |  |  |  |
| 3 | 1 g-180 °C-10 h | 3.73 % |  |  |  |
| 4 | 1 g-200 °C-12 h | 2.30 % |  |  |  |
| 5 | 2 g-140 °C-8 h | 2.95 % | 3.15 % | 2.36 % | 2.39 % |
| 6 | 2 g-160 °C-6 h | 2.74 % |  |  |  |
| 7 | 2 g-180 °C-12 h | 2.34 % |  |  |  |
| 8 | 2 g-200 °C-10 h | 4.56 % |  |  |  |
| 9 | 3 g-140 °C-10 h | 1.53 % | 2.60 % | 2.21 % | 3.00 % |
| 10 | 3 g-160 °C-12 h | 3.13 % |  |  |  |
| 11 | 3 g-180 °C-6 h | 1.72 % |  |  |  |
| 12 | 3 g-200 °C-8 h | 4.01 % |  |  |  |
| 13 | 4 g-140 °C-12 h | 2.23 % | 1.67 % | 3.08 % | 2.50 % |
| 14 | 4 g-160 °C-10 h | 2.00 % |  |  |  |
| 15 | 4 g-180 °C-8 h | 1.03 % |  |  |  |
| 16 | 4 g-200 °C-6 h | 1.43 % |  |  |  |
| Range R | Factor A  (Amount of quinoa saponin powder)  1.48 % | | Factor B  (Temperature)  1.07 % | | Factor C  (Time)  1.2 % |
| Primary and secondary order | | | A＞C＞B | | |
| Optimal combination of orthogonal design | | | 2 g - 200 °C - 10 h | | |

Note: k_Ai (i=1-4)_ denotes the average of the results of each level of feeding volume (1 g, 2 g, 3 g, 4 g), respectively. k_Bi (i=1-4)_ denotes the average of the results of each level of reaction temperature (140 °C，160 °C，180 °C，200 °C), respectively. k_Ci (i=1-4)_ denotes the mean of the results for each level of reaction time (6 h, 8 h, 10 h, 12 h), respectively. R is defined as the range between the maximum and minimum value of k_j,i_ (j=A,B,C; i=1-4) and is used for evaluating the importance of the factors and the levels, The typical calculation process is shown below:

Take the calculation of factor B for example:

| $\text{k}_{\text{B1}}=\frac{QY_{1}+QY_{5}+QY_{9}+QY_{13}}{4}=\frac{(1.32+2.95+1.53+2.23)\%}{4}=2.01\%$ | (1) |
| --- | --- |
| $\text{k}_{\text{B2}}=\frac{QY_{2}+QY_{6}+QY_{10}+QY_{14}}{4}=\frac{(1.58+2.74+3.13+2.00)\%}{4}=2.\text{36 }\%$ | (2) |
| $\text{k}_{\text{B3}}=\frac{QY_{3}+QY_{7}+QY_{11}+QY_{15}}{4}=\frac{(3.73+2.34+1.72+1.03)\%}{4}=2.21\%$ | (3) |
| $\text{k}_{\text{B4}}=\frac{QY_{4}+QY_{8}+QY_{12}+QY_{16}}{4}=\frac{(2.30+4.56+4.01+1.43)\%}{4}=3.08\%$ | (4) |
| $\text{R}_{\text{B}}=\text{ma}x_{k_{B4}}-\min_{k_{B1}}=(3.08-2.01)\%=1.07\%$ | (5) |

1. ***Optimization of doping conditions***

According to the above optimization results, others 3-factor (basic conditions, nitrogen dopant type and nitrogen dopant amount), 4-level orthogonal experiments were designed, as shown in Table S4. The orthogonal table L_16_ (4^3^) as shown in Table S5 was used to conduct the orthogonal experiments. A total of 16 sets of orthogonal experiments were designed, for which the FL quantum yields were measured, and the results are shown in Table S6. The results of data analysis showed that the optimum synthetic conditions should be: 2 g quinoa saponin powder, 2.668 mL (0.04 mol) EDA, hydrothermal temperature of 200 °C, and hydrothermal time of 10 h. The QY of the obtained CQDs was 22.24 %. The synthesis conditions have good repeatability (RSDs = 1.29 %), as shown in Figure S1.

**Table S4.** The factors and levels of orthogonal experiment (doping conditions).

| Level  Factors | A  Basic conditions | B  Type of dopant | C  Amount of dopant / mol |
| --- | --- | --- | --- |
| 1 | No. 3 (1 g-180 °C-10 h) | Ethylenediamine (EDA) | 0.02 mol |
| 2 | No. 8 (2 g-200 °C-10 h) | Carbamide (Urea) | 0.04 mol |
| 3 | No. 10 (3 g-160 °C-12 h) | Aqueous ammonia (NH_4_OH, 25 %) | 0.06 mol |
| 4 | No. 12 (3 g-200 °C-8 h) | Diethanolamine (DEA) | 0.08 mol |

**Table S5.** The orthogonal experiments scheme (doping conditions).

| Test  number  Factors | A  Basic conditions | B  Type of dopant | C  Amount of dopant / mol |
| --- | --- | --- | --- |
| 1 | No. 3 (1 g-180 °C-10 h) | EDA | 0.02 mol |
| 2 | No. 3 (1 g-180 °C-10 h) | Urea | 0.04 mol |
| 3 | No. 3 (1 g-180 °C-10 h) | NH_4_OH | 0.06 mol |
| 4 | No. 3 (1 g-180 °C-10 h) | DEA | 0.08 mol |
| 5 | No. 8 (2 g-200 °C-10 h) | EDA | 0.04 mol |
| 6 | No. 8 (2 g-200 °C-10 h) | Urea | 0.02 mol |
| 7 | No. 8 (2 g-200 °C-10 h) | NH_4_OH | 0.08 mol |
| 8 | No. 8 (2 g-200 °C-10 h) | DEA | 0.06 mol |
| 9 | No. 10 (3 g-160 °C-12 h) | EDA | 0.06 mol |
| 10 | No. 10 (3 g-160 °C-12 h) | Urea | 0.08 mol |
| 11 | No. 10 (3 g-160 °C-12 h) | NH_4_OH | 0.02 mol |
| 12 | No. 10 (3 g-160 °C-12 h) | DEA | 0.04 mol |
| 13 | No. 12 (3 g-200 °C-8 h) | EDA | 0.08 mol |
| 14 | No. 12 (3 g-200 °C-8 h) | Urea | 0.06 mol |
| 15 | No. 12 (3 g-200 °C-8 h) | NH_4_OH | 0.04 mol |
| 16 | No. 12 (3 g-200 °C-8 h) | DEA | 0.02 mol |

Note: The factors A (No. 3, No. 8, No. 10, and No. 12) were selected from the optimal basic conditions.

**Table S6.** The numerical of orthogonal tests (synthetic conditions).

| Test number | Experimental combinations | QY / % | k_Ai_ _(i=1-4)_ | k_Bi (i=1-4)_ | k_Ci (i=1-4)_ |
| --- | --- | --- | --- | --- | --- |
| 1 | EDA 0.02 mol，1 g-180 °C-10 h | 14.00 % | 9.59 % | 16.49 % | 10.71 % |
| 2 | EDA 0.04 mol，2 g-200 °C-10 h | 22.24 % |  |  |  |
| 3 | EDA 0.06 mol，3 g-160 °C-12 h | 14.45 % |  |  |  |
| 4 | EDA 0.08 mol，3 g-200 °C-8 h | 15.27 % |  |  |  |
| 5 | Urea 0.02 mol，2 g-200 °C-10 h | 14.31 % | 12.23 % | 11.72 % | 11.73 % |
| 6 | Urea 0.04 mol，1 g-180 °C-10 h | 9.77 % |  |  |  |
| 7 | Urea 0.06 mol，3 g-200 °C-8 h | 11.33 % |  |  |  |
| 8 | Urea 0.08 mol，3 g-160 °C-12 h | 11.46 % |  |  |  |
| 9 | NH_4_OH 0.02 mol，3 g-160 °C-12 h | 9.43 % | 9.93 % | 9.32 % | 9.89 % |
| 10 | NH_4_OH 0.04 mol，3 g-200 °C-8 h | 10.55 % |  |  |  |
| 11 | NH_4_OH 0.06 mol，1 g-180 °C-10 h | 9.35 % |  |  |  |
| 12 | NH_4_OH 0.08 mol，2 g-200 °C-10 h | 7.95 % |  |  |  |
| 13 | DEA 0.02 mol，3 g-200 °C-8 h | 5.11 % | 10.57 % | 4.79 % | 9.98 % |
| 14 | DEA 0.04 mol，3 g-160 °C-12 h | 4.36 % |  |  |  |
| 15 | DEA 0.06 mol，2 g-200 °C-10 h | 4.43 % |  |  |  |
| 16 | DEA 0.08 mol，1 g-180 °C-10 h | 5.25 % |  |  |  |
| Range R | Factor A  (Basic conditions)  2.64 % | Factor B  (Type of dopant) 11.70 % | | Factor C  (Amount of dopant) 1.84 % | |
| Primary and secondary order | | | B＞A＞C | | |
| Optimal combination of orthogonal design | | | EDA 0.04 mol, 2 g-200 °C-10 h | | |

Note: k_Ai (i=1-4)_ denotes the mean of the results for each level of the base conditions (basic conditions for No. 3, No. 8, No. 10, and No. 12), respectively. k_Bi (i=1-4)_ denotes the average of the results for each level of dopant type (EDA, Urea, NH_4_OH, DEA), respectively. k_Ci (i=1-4)_ denotes the average of the results for each level of dopant addition (0.02 mol, 0.04 mol, 0.06 mol, 0.08 mol), respectively. R is defined as the range between the maximum and minimum value of k_j,i_ (j=A,B,C; i=1-4) and is used for evaluating the importance of the factors and the levels, The typical calculation process is shown below:

Take the calculation of factor A for example:

| $\text{k}_{\text{A1}}=\frac{QY_{1}+QY_{6}+QY_{11}+QY_{16}}{4}=\frac{(14+19.77+9.35+5.25)\%}{4}=9.59\%$ | (6) |
| --- | --- |
| $\text{k}_{\text{A2}}=\frac{QY_{2}+QY_{5}+QY_{12}+QY_{15}}{4}=\frac{(22.24+14.31+7.95+4.43)\%}{4}=\text{12.23 }\%$ | (7) |
| $\text{k}_{\text{A3}}=\frac{QY_{3}+QY_{8}+QY_{9}+QY_{14}}{4}=\frac{(14.45+11.46+9.43+4.36)\%}{4}=\text{9.93 }\%$ | (8) |
| $\text{k}_{\text{A4}}=\frac{QY_{4}+QY_{7}+QY_{10}+QY_{13}}{4}=\frac{(15.27+11.33+10.55+5.11)\%}{4}=10.57\%$ | (9) |
| $\text{R}_{\text{A}}=\text{ma}x_{k_{A2}}-\min_{k_{A1}}=(12.23-9.59)\%=2.64\%$ | (10) |


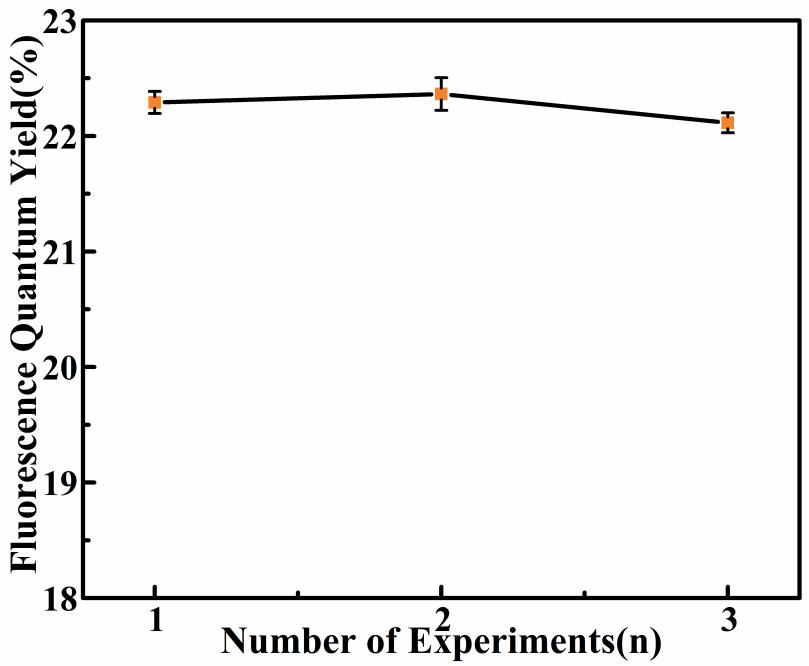


Figure S1. Repeatability test of synthesis conditions.
